# A survey of Irish red foxes (*Vulpes vulpes*) to establish the prevalence of *Mycobacterium bovis*

**DOI:** 10.1186/s13620-025-00295-2

**Published:** 2025-03-20

**Authors:** Denise Murphy, William FitzGerald, Guy McGrath, Catherine Swan, Kevin Kenny

**Affiliations:** 1 Department of Agriculture, Food and the Marine, Athlone Regional Veterinary Laboratory, Coosan, Athlone, Co Westmeath Ireland; 2 Department of Agriculture, Food and the Marine, Backweston Campus, Stacumny Lane, Celbridge Co., Kildare, Ireland; 3 Centre for Veterinary Epidemiology and Risk Analysis, UCD School of Veterinary Medicine, University College Dublin, Belfield, Dublin 4, Ireland; 4 Department of Agriculture, Food and the Marine Laboratories, Backweston Campus, Stacumny Lane, Celbridge Co., Kildare, Ireland

**Keywords:** *Mycobacterium bovis*, Ireland, Red fox, Bovine tuberculosis, Wildlife risk

## Abstract

Between October 2018 and December 2020, an opportunistic collection of tissues from 218 foxes was undertaken to investigate the prevalence of *Mycobacterium bovis (M. bovis)* in this species. A pooled sample of lymph nodes, lung and other tissues from each fox, was cultured for the presence of *M. bovis.* The organism was not isolated from any fox samples, but non-tuberculous mycobacteria were recovered from 20 foxes. These results suggest that it is unlikely that foxes represent a significant wildlife source of *M. bovis* in Ireland.

## Background

*Mycobacterium bovis* causes Bovine tuberculosis (bTB), which is controlled under Ireland’s statutory eradication programme. The disease is also recorded in other European countries (France, Italy, Portugal, Spain, United Kingdom) and while cattle are the primary hosts of *M. bovis*, this pathogen has a broad host range [1] and several wildlife species may act as reservoirs of this bacterium [2]. Badgers (*Meles meles*) have been identified as playing a role in the transmission of the disease in Ireland [3] while wild boar (*Sus scrofa*) are a reservoir host in the Iberian Peninsula [4]. Across Europe, deer species are known to act as sources of infection for cattle, in localised areas of high density [5, 6]. The brushtail possum (*Trichosurus vulpecula*, New Zealand), the African buffalo (*Syncerus caffer*, South Africa) and the white-tailed deer (*Odocoileus virginianus,* USA) are other known wildlife reservoirs of *M. bovis*. Transmission at a cattle-wildlife interface will reflect wildlife disease prevalence and population density, habitat overlap and contact between the species. Wildlife acting as a maintenance host with spillback to cattle, pose substantial challenges to bTB disease eradication programmes [7].

In recent years, the potential role of other wild mammals in the epidemiology of bTB has been examined. *M. bovis* infection has been reported in red foxes (*Vulpes vulpes*) in Spain [8], the United Kingdom [5], Portugal [9] and France [10]. The estimated prevalence of *M. bovis* amongst fox populations in these studies varies from 3.17% in the UK to 26.9% in Portugal. Possible modes of infection include feeding on infected wild animal carcases and close interaction with infected badgers, cattle or a contaminated environment. The prevalence of tuberculosis in Irish foxes has not been studied to date. However, foxes are sampled on an annual basis by the Department of Agriculture, Food and the Marine (DAFM), as part of a surveillance programme to prove the absence of the zoonotic tapeworm *Echinococcus multilocularis* from the fox population [11]. Sampling of a subset of this fox group for mycobacterial culture was undertaken to allow the prevalence of *M. bovis* infection in Irish foxes to be estimated.

## Materials and methods

Each year, with the assistance of the National Association of Regional Game Councils, DAFM arranges the collection of approximately 400 Irish red foxes shot by licenced hunters. Carcases are submitted to a Regional Veterinary Laboratory for sampling as part of the *Echinococcus multilocularis* surveillance programme. Foxes are sourced from across the 26 counties of the Republic of Ireland and the shooting locations are recorded at District Electoral Division (DED) level. The foxes for the current study were sourced from gun clubs and represent a sample of convenience using the existing *Echinococcus multilocularis* survey to obtain the tissues. Using the Ausvet epidemiological calculators and estimating a fox *M. bovis* infection prevalence of 3%, a test sensitivity of 0.5, a test specificity of 1 and with a desired confidence of 0.95, the subset to be sampled for mycobacterial culture was calculated to be a minimum of 91 foxes [12].

For the selected foxes, lymph nodes (parotid, retropharyngeal, submandibular, prescapular, mediastinal, bronchial, hepatic, mesenteric, inguinal and popliteal), lung, spleen, liver and kidney were examined macroscopically and sampled to create a tissue pool for mycobacterial culture. The composite tissue sample for each fox was placed in a screw top sterile container and frozen at −20°C.

In advance of culture, each tissue pool was thawed at room temperature and then homogenised in saline. The homogenate was decontaminated in oxalic acid and after rinsing, the pellet was used to inoculate a BACTEC™ Mycobacteria Growth Indicator Tube, a slope of Lowenstein-Jensen medium supplemented with pyruvate and a slope of modified 7H11 media supplemented with pyruvate [13]. The incubation period was 7 weeks and Ziehl–Neelsen staining was performed on smears prepared from cultures which displayed growth. Acid-fast bacilli obtained from liquid or solid media were tested using a real-time PCR to ascertain if the isolates were members of the *Mycobacterium tuberculosis* complex or were non-tuberculous mycobacteria [13].

## Results

**Table:** 

*Key findings summary*:
• 218 foxes sampled- no *M. bovis* detected
• Sampling covered 24 of 26 Irish counties
• Non-tuberculous mycobacteria isolated from 20 foxes in 14 counties

**Fig. 1:**
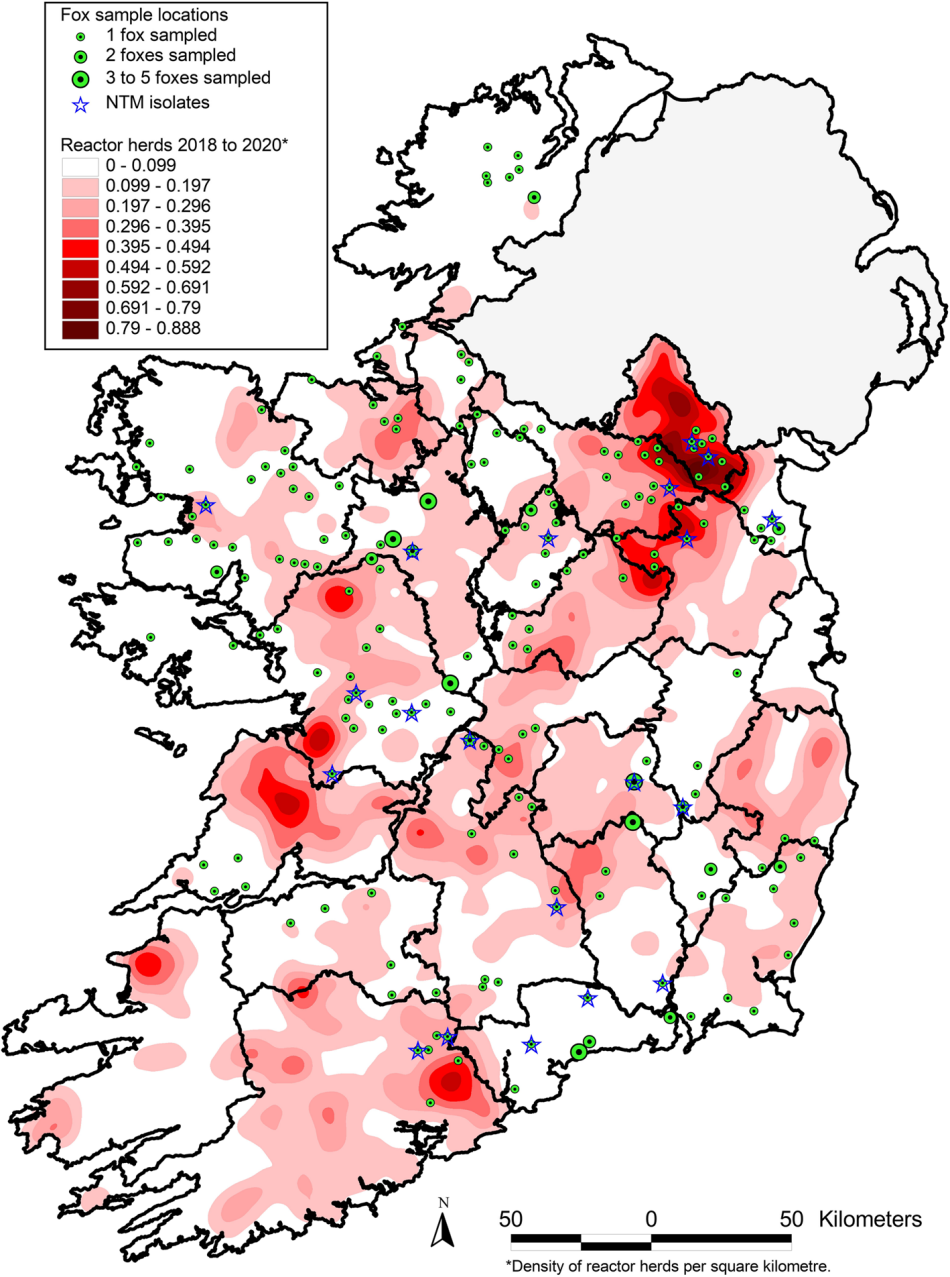
Map illustrating the locations of foxes examined and the non-tuberculous mycobacteria isolates plotted against a kernel density map (kernel bandwidth 10 km, grid size 100 m) of herds that had a breakdown of bovine TB in Ireland 2018–2020

Tissues for mycobacterial culture were collected from foxes in a six-week period from late October to early December. There were 170 foxes sampled in 2018 with a further 48 animals sampled in 2020. These 218 foxes originated from 24 different counties in the Republic of Ireland. Gross lesions suspicious of tuberculosis were not seen in any of the examined tissues. Of the 218 tissue pools cultured, 198 did not yield any mycobacterial isolate while non-tuberculous mycobacteria (NTM) were recovered from 20 foxes located in 14 different counties of the 26 counties in the Republic of Ireland. The breakdown of the numbers examined and the number of the NTM isolates from each county and province is shown in Table 1. The locations of foxes tested for tuberculosis highlighting the NTM isolates, was plotted against a kernel density surface of herds that had a bTB breakdown in Ireland between 2018–2020 (Fig. 1).

**Table 1:** The number of foxes sampled for mycobacterial culture and the number with non-tuberculous mycobacteria isolates, from the counties and provinces in Ireland 2018–2020

Province	County	No. of foxes cultured (non-tuberculous mycobacteria)
*Munster*		
	Clare	4(0)
	Cork	6 (2)
	Kerry	0 (0)
	Limerick	6 (0)
	Tipperary	11 (2)
	Waterford	10 (2)
		**37 (6)**
*Leinster*		
	Carlow	1(0)
	Dublin	0(0)
	Kildare	5(1)
	Kilkenny	6(1)
	Laois	4(1)
	Longford	10(1)
	Louth	7(1)
	Meath	6(1)
	Offaly	5(0)
	Westmeath	6(0)
	Wexford	14(0)
	Wicklow	3(0)
		**67(6)**
*Connacht*		
	Galway	24(3)
	Mayo	20(1)
	Leitrim	9(0)
	Roscommon	15(1)
	Sligo	7(0)
		**75(5)**
*Ulster*		
	Cavan	22(1)
	Donegal	8(0)
	Monaghan	9(2)
		**39(3)**
**TOTAL**		**218(20)**

## Discussion

This is the first survey of *M. bovis* prevalence in red foxes in Ireland and the results suggest this species is not a significant host of *M. bovis* and does not represent a substantial risk for the spread of this organism to the Irish cattle population. While using an existing survey to obtain the samples, wide geographic coverage was attained with foxes examined from 24 of the 26 counties in the Republic of Ireland. When the locations of the sampled foxes are mapped against bTB hotspot areas there is considerable overlap between the two, particularly in the north-east counties which have a high prevalence of bTB. Given the potential modes of exposure of foxes to *M. bovis*, there should be a greater likelihood of detecting infection in these foxes due to spillover infection if it were happening.

A potential weakness in the survey is that 34.4% of foxes sampled were in Connaught, a province in the west where cattle densities and herd level incidence of bTB are low (~ 2.83%), while 17% of foxes cultured were from Munster, the province with the greatest density of cattle and a higher herd level prevalence (~ 5.26%). Despite this variation in bTB prevalences in cattle within the regions from which foxes were sampled, a general interpretation can be made that the overall national risk the red fox poses to the spread of bTB in Ireland appears low.

A recent study in France examined 568 foxes from four TB endemic areas for *M. bovis* [10]. Infected foxes were found in three different study areas in southwest France with prevalence rates of 5%, 7.1% and 9.2%. These fox infection rates are similar to those recorded for badgers and wild boar in the same three areas, suggesting a role in TB epidemiology is possible. For the fourth study area in Burgundy, *M. bovis* infection was not detected in sampled foxes, and the prevalence of bTB in cattle had decreased in this area. The value of testing the mesenteric lymph nodes is evident with infection recorded in this tissue for 68% of foxes deemed to be infected.

The isolation of non-tuberculous mycobacteria (NTM) from 20 foxes is not unexpected. The isolation of NTM indicates the ability of the culture technique used in the survey to isolate *Mycobacterium* species. NTM are ubiquitous in the environment and are found in soil and water [14]. NTM have been isolated from several wildlife species [15]. *M. avium* has been isolated from wild boar [16] and from roe deer (*Capreolus capreolus*) and foxes in Spain [17]. *M. avium* subsp. *hominissuis* has been reported in wild boar in Portugal [18]. The significance of the isolation of NTM in foxes is unclear; we did not speciate the isolates as it was not part of this study. Speciation of the NTM isolates from this study could be undertaken along with NTM from other wildlife e.g. badgers as part of further studies on NTM in wildlife in Ireland.

Although there was no evidence of *M. bovis* infection in the foxes sampled in this survey, it is important to remember that the presence of infection in a wild animal population is not, of itself, evidence that this species is a wildlife reservoir host for tuberculosis. The pathology and epidemiology of TB in wildlife, differs depending on the species affected and their environment [19] and this species-specific information must be considered, along with the prevalence of infection, when assessing the significance of an infected wild animal species as a reservoir host for tuberculosis and the potential risks of transmission to cattle [2]. In the UK study, a semi-quantitative risk assessment showing the potential risk of disease transmission from foxes to cattle, relative to the badger, demonstrated that the risk was not significant [5].

It is possible for this survey to be repeated at intervals in the future as foxes are tested annually in Ireland for the *Echinococcus multilocularis* surveillance programme. Improvements to the spatial distribution of fox sampling locations could be addressed in any future surveys and the value of additional culture of individual tissues such as the mesenteric lymph nodes examined. Several modes of infection of foxes have been hypothesised in previous studies, including feeding on infected carcases of wild animals [20], sharing or inhabiting disused badger setts [5] or close interaction with infected cattle herds and their facilities [21]. Previous studies have shown that *M. bovis* circulates in cattle, badgers and deer in County Wicklow [6], and foxes may have access to deer tissue discarded by hunters, to disused badger setts or to badgers killed on roads, therefore a single study of infection of foxes in this location has merit.

## Data Availability

No datasets were generated or analysed during the current study.

## Abbreviations

bTB: Bovine tuberculosis
DAFM: Department of Agriculture, Food and the Marine
DED: District Electoral Division
NTM: Non-tuberculous mycobacteria
TB: Tuberculosis
